# Comparative transcriptome analysis coupled to X-ray CT reveals sucrose supply and growth velocity as major determinants of potato tuber starch biosynthesis

**DOI:** 10.1186/1471-2164-11-93

**Published:** 2010-02-05

**Authors:** Stephanus J Ferreira, Melanie Senning, Sophia Sonnewald, Petra-Maria Keßling, Ralf Goldstein, Uwe Sonnewald

**Affiliations:** 1 Friedrich-Alexander-University Erlangen-Nuremberg, Department of Biology, Staudtstrasse 5, 91058 Erlangen, Germany; 2 Fraunhofer Institute for Integrated Circuits IIS, Development Centre X-Ray Technology, Dr-Mack-Straße 81, 90762 Fürth, Germany; 3 Fraunhofer Institute for Integrated Circuits IIS, Contactless Test and Measuring Systems, Am Wolfsmantel 33, 91058 Erlangen, Germany

## Abstract

**Background:**

Even though the process of potato tuber starch biosynthesis is well understood, mechanisms regulating biosynthesis are still unclear. Transcriptome analysis provides valuable information as to how genes are regulated. Therefore, this work aimed at investigating transcriptional regulation of starch biosynthetic genes in leaves and tubers of potato plants under various conditions. More specifically we looked at gene expression diurnally in leaves and tubers, during tuber induction and in tubers growing at different velocities. To determine velocity of potato tuber growth a new method based on X-ray Computed Tomography (X-ray CT) was established.

**Results:**

Comparative transcriptome analysis between leaves and tubers revealed striking similarities with the same genes being differentially expressed in both tissues. In tubers, oscillation of granule bound starch synthase (GBSS) expression) was observed which could be linked to sucrose supply from source leaves. X-ray CT was used to determine time-dependent changes in tuber volume and the growth velocity was calculated. Although there is not a linear correlation between growth velocity and expression of starch biosynthetic genes, there are significant differences between growing and non-growing tubers. Co-expression analysis was used to identify transcription factors positively correlating with starch biosynthetic genes possibly regulating starch biosynthesis.

**Conclusion:**

Most starch biosynthetic enzymes are encoded by gene families. Co-expression analysis revealed that the same members of these gene families are co-regulated in leaves and tubers. This suggests that regulation of transitory and storage starch biosynthesis in leaves and tubers, respectively, is surprisingly similar. X-ray CT can be used to monitor growth and development of belowground organs and allows to link tuber growth to changes in gene expression. Comparative transcriptome analysis provides a useful tool to identify transcription factors possibly involved in the regulation of starch biosynthesis.

## Background

Starch is not only the most important carbohydrate source to the human diet, but has major industrial applications. It consists of two major fractions, amylose and amylopectin. Amylose is essentially a linear polymer of glucose units linked with alpha (1,4) bonds whilst amylopectin has a higher percentage of branched alpha (1,6) bonds. Due to its importance, crop plants producing starch in large quantities have been extensively researched. The fourth most important crop in the world in terms of total biomass produced is potato and this is due to its starchy tuber which can store up to 80% of its dry weight as starch [1].

In potato starch is either accumulated transiently in leaves or as storage starch in tubers. In leaves starch is synthesised in the chloroplast from triose-phosphates produced during photosynthesis. After several intermediate steps, glucose 6-phosphate is converted to glucose 1-phosphate by the enzyme phosphoglucomutase (PGM). Glucose 1-phosphate, along with ATP serves as substrate for ADP-glucose production by ADP-glucose pyrophosphorylase (AGPase), which is the first reaction committed to starch biosynthesis. ADP-glucose is then the glycosyl donor for the various starch synthases forming linear glucans which are subsequently branched by branching enzymes to produce starch.

For starch production in the tuber photoassimilates, in the form of sucrose, must first be imported from photoautotrophic tissue via the phloem [2]. There is conflicting evidence to whether carbon accumulation is sink or source limited. It has been shown that under normal conditions flux control of tuber starch biosynthesis is mostly source regulated [3]. In another study, reduced photosynthetic activity of potato leaves by silencing cytosolic fructose 1, 6 bisphosphatase did not have an effect on tuber yield or plant growth and it was concluded that tuber starch biosynthesis is not source limited [4]. Both studies however emphasize the importance of sucrose supply to the tuber. This idea is further supported by constitutive [5] or phloem-specific [6] antisense inhibition of the sucrose transporter. Sucrose transport activity is essential for apoplastic phloem loading, hence, silencing its expression lead to a reduced phloem loading of sucrose which was accompanied by decreased photosynthetic rates and reduced tuber yield of transgenic potato plants. In another study [7], over-expression of a sucrose transporter from spinach in potato lead to a reduced sucrose level in leaves and an increased sucrose content of tubers. However, this had no effect on tuber starch content. Even though this did not lead to an increased starch content of tubers, it did provide evidence as to the important role of sucrose as regulator of carbon metabolism [7]. Of particular note was the decrease in plastidial amino acid synthesis even though sucrose was still in abundant supply.

There is also conflicting evidence as to whether the supply rate of sucrose from the leaves to tubers is constant throughout the diurnal cycle or whether there is a diurnal rhythm. It has been shown that source to sink carbon flux is constant [8]. It can also be argued that for starch biosynthesis in tubers to be unaffected by limitations in photosynthetic capacity [4], sucrose supply to the tuber should be constant. It has been shown that there are significant differences of tuber sucrose content at the start and end of the light period and that this has an effect on tuber metabolism [9].

**Figure 1:**
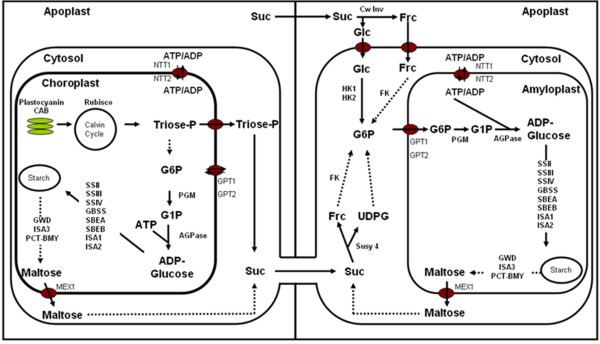
**Proposed pathway of starch metabolism in photosynthetic and non-photosynthetic tissue**.

After the formation of tubers they represent the predominant sink in the plant. The onset of tuberisation is marked by many physiological and biochemical changes in the stolons. The major changes which are seen as markers of tuberisation are the switch from apoplastic to symplastic sucrose unloading and the change from hydrolytic to sucrolytic cleavage of sucrose [10]. Sucrose synthase (Susy) activity increases with the development of the tuber and is the major determinant of potato sink strength [11]. After the cleavage of sucrose to UDP-glucose and fructose by Susy, the two molecules must be converted to glucose 6-phosphate via different pathways for starch biosynthesis. The major difference between starch biosynthesis in tubers compared to leaves is that hexose-phosphate, in the form of glucose 6-phosphate, and ATP must be imported from the cytosol. Although there is evidence that glucose 1-phosphate can also be transported across the plastid membrane and directly used for starch biosynthesis via starch phosporylase in potato tuber discs [12], it seems that *in vivo *glucose 6-phosphate is the predominant form of hexose-phosphate transport across the amyloplast membrane. Transport of glucose 6-phosphate and ATP across the amyloplast membrane is facilitated by the glucose 6-phosphate translocator (GPT) [13], and the plastidial ATP/ADP translocator (NTT) [14], respectively. Simultaneous over-expression of these transporters lead to an increase in total starch yield per plant showing that starch biosynthesis is co-limited by hexose-phosphate and ATP in the plastid [15]. Glucose 6-phosphate is then converted to glucose 1-phosphate by plastidial PGM. Silencing of plastidial PGM lead to dramatic reduction in starch content of the tubers, illustrating not only the importance of this enzyme in starch biosynthesis, but provides compelling evidence that starch is synthesised from imported glucose 6-phosphate [16]. As mentioned earlier, glucose 1-phosphate and ATP serve as substrates for ADP-glucose synthesis by AGPase and further starch biosynthesis is similar to leaves. Since large scale comparative transcriptome analysis comparing leaf and tuber starch biosynthesis have not been done, it is not known whether or not the same isoforms of these genes are active in both tissues. The importance of AGPase in starch biosynthesis has been shown on various occasions and reduced activity lead to low starch, high sucrose containing tubers [17]. As far as increasing starch biosynthesis by over-expression of AGPase is concerned, two studies over-expressing an *E. coli *AGPase give conflicting results. Stark *et al. *[18] could increase starch content by over-expression but Sweetlove *et al. *[19] showed that increased biosynthesis is accompanied by increased breakdown. There are mainly four soluble starch synthase enzymes responsible for biosynthesis, starch synthase I, II, III, IV and one that is exclusively granule bound, named granule bound starch synthase (GBSS). Soluble starch synthases and the branching enzymes are believed to be responsible for amylopectin synthesis. The major starch synthases are starch synthase II and III and silencing of these two isoforms lead to greatly altered amylopectin structure [20]. Granule bound starch synthase is exclusively responsible for the synthesis of amylose and a potato mutant lacking any activity produce amylose free starch [21]. Starch synthase IV seems to be important for starch granule initiation and mutants lacking this isoform do not only show changes in diurnal starch accumulation, but also have fewer, and bigger, starch granules [22,23]. Despite the many isoforms of branching enzyme, they can be grouped in starch branching enzyme A (SBEA) and B (SBEB). The majority of branching enzyme activity can be attributed to SBEB [24], but silencing of only SBEB did not lead to a change in starch structure. Simultaneous silencing of SBEA and SBEB, however, lead to production of high amylose potato starch [25]. Table 1 contains all the genes involved in starch metabolism which will be further discussed in this paper. The pathways in which these enzymes are involved are shown in figure 1.

**Table 1:** List of genes involved in starch metabolism discussed in this paper.

Name	Abbreviated name	POCI identifier
Plastocyanin	Plastocyanin	Micro.4322.c1
Chlorophyll a/b binding	CAB	Micro.4163.c1
Ribulose 1,5 bisphosphate carboxylase oxygenase	Rubisco	Micro.4165.c3
Triose phosphate translocator 1	TPT	Micro.3160.c1
Glucose 6-phosphate translocator 1	GPT1	Micro.4029.c2
Glucose 6-phosphate translocator 2	GPT2	Micro.1076.c1
Plastidial phosphoglucomutase	PGM	Micro.1743.c2
ATP/ADP translocator 1	NTT1	Micro.1831.c2
ADP-glucose pyrophosphorylase large subunit	AGPase LS	Micro.2198.c1
ADP-glucose pyrophosphorylase small subunit	AGPase SS	Micro.367.c1
Starch synthase II	SSII	Micro.1850.c2
Starch synthase III	SSIII	Micro.1658.c1
Granule bound starch synthase	GBSS	Micro.920.c2
Starch synthase IV	SSIV	Micro.16059.c1
Starch branching enzyme A	SBEA	Micro.16220.c1
Starch branching enzyme B	SBEB	Micro.1689.c1
Isoamylase 1	ISA1	Micro.7513.c1
Isoamylase 2	ISA2	Micro.13258.c1
Glucan, water dikinase	GWD	Micro.3453.c1
Isoamylase 3	ISA3	Micro.10651.c1
PCT-Beta-amylase	PCT-BMY	Micro.13823.c2
Maltose transporter	MEX1	Micro.10450.c1
Alpha-amylase	AAMY	Micro.10377.c1
Disproportionating enzyme 1	DPE1	Micro.1834.c1
Disproportionating enzyme 2	DPE2	Micro.6841.c1
SEX4 phosphoglucan phosphatase	SEX4	Micro.1811.c1
Cell wall invertase	Cw-Inv	Micro.4223.c2
Fructokinase	FK	Bf_suspxxxx_0040h09.t3m.scf
Sucrose synthase 4	Susy4	Micro.196.c8
Hexokinase 1	HK1	Micro.5594.c1
Hexokinase 2	HK2	Micro.4130.c1

One of the major problems with potato tuber research is the fact that tuber growth and induction rates are not synchronised. Although tuber developmental stages are well defined [26], this does not mean that tubers in the same stage have similar growth- or biochemical characteristics. Adding to this, tubers are underground organs making *in vivo *analysis without damaging the plant almost impossible. To date no study has determined the *in vivo *growth velocity of individual tubers. X-ray computed tomography (X-ray CT) provides the opportunity to determine the velocity of tubers without physically damaging the plant. It has been used to study underground plant organs [27], but to date no study has determined tuber growth velocity using this method.

Even though the pathway of starch biosynthesis is well understood, mechanisms regulating biosynthesis are still unclear. Smith *et al. *[28] did an extensive study on transcriptional regulation of starch metabolism in Arabidopsis leaves over a 24 hour diurnal period. This study was very useful elucidating the regulating machinery of starch metabolism. The design of a custom microarray has made it possible to do similar types of analysis in potato. The POCI microarray was designed from the largest collection of ESTs from potato yet [29] and can be used to study all aspects of the potato transcriptome, which include starch biosynthesis. Due to the abundance of transcriptome data available, co-expression analysis has become a common method to identify new genes involved in specific metabolic pathways. The principle of the technique is that genes involved in similar processes would be expressed or inhibited at the same time point or under similar conditions. This would not only include structural genes, but possibly also regulatory genes like transcription factors. They are able to bind to specific sequences of various targets and thus have the ability to regulate entire metabolic pathways [30]. Moreover, over-expression of two transcription factors in tomato led to the over-expression of the entire anthocyanin synthesis pathway, producing purple anthocyanin rich tomatoes [31]. These results show that by manipulating transcription factors entire biosynthetic pathways can be influenced.

The aim of this work was to investigate the transcriptional regulation of starch biosynthesis in potato under various conditions. More specifically we looked at similarities between gene expression in leaves and tubers. Furthermore we established a new technique using X-ray computed tomography to determine tuber growth velocity *in vivo *and used it to analyse gene expression in tubers growing at different velocities. Finally comparative analysis of transcription profiles were used to identify transcription factors possibly regulating starch biosynthesis.

## Results and discussion

### Starch biosynthesis in potato leaves follow carbohydrate accumulation and show similarities to tuber starch biosynthesis

**Figure 2:**
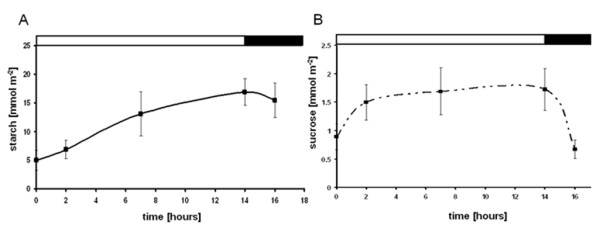
**Diurnal starch and sucrose content of leaves over a sixteen hour period**. A) Starch and B) sucrose content. Error bars indicate standard deviation (n = 3).

Leaf samples for starch and RNA extractions were taken from potato plants grown under a 14 hour light and 10 hour dark cycle. The light conditions were chosen to ensure diurnal turnover of transitory starch. As was expected, starch levels were the lowest at the start of the light period and accumulated during the day to a high at the end of the light period (Figure 2A). The same pattern was observed for sucrose although the accumulation and decline was more rapid (Figure 2B). The levels of starch and sucrose compared well to what was previously measured in potato leaves [4].

**Figure 3:**
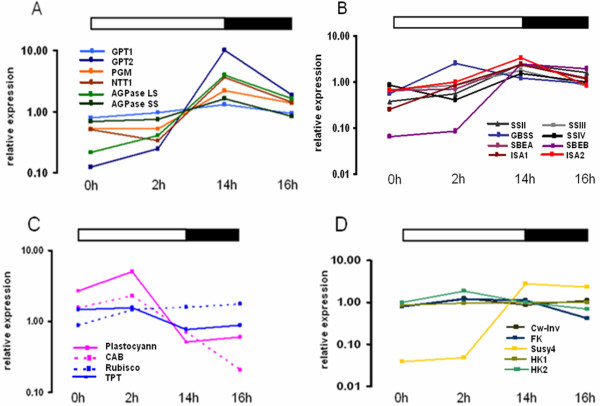
**Diurnal expression of genes known to be involved in starch biosynthesis**. A) Import of glucose 6-phosphate and ATP into the plastid and the conversion thereof to ADP-Glucose. GPT1 (light blue), GPT2 (dark blue), PGM (orange), NTT1 (brown), AGPase LS (light green), AGPase SS (dark green). B) Starch synthases and branching enzymes. SSII (dark grey), SSIII (light grey), GBSS (blue), SSIV (black), SBEA (light purple), SBEB (dark purple), ISA1 (dark red) and ISA2 (pink). C) Photosynthetic and Calvin cycle related genes. Plastocyanin (pink solid), CAB ( pink dotted), Rubisco (blue dotted), TPT (blue solid). D) Sucrose cleavage and phosphorylation. CW-Inv (olive green), FK (blue), Susy (gold), HK1 (dark yellow) and HK2 (green) Values are the mean of two replicates.

**Figure 4:**
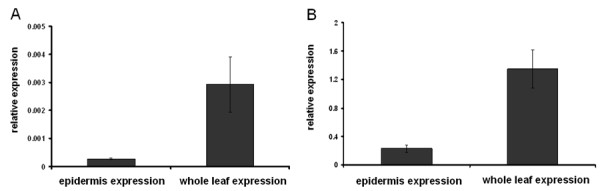
**Relative expression of GPT and NTT in epidermal and whole leaf tissue**. A) GPT and B) NTT. Error bars represent standard deviation (n = 3).

After confirming that there was a turnover of transient starch under the specific light conditions used, gene expression at different times of the day was analysed by microarray. To investigate how much similarity there is between leaf and tuber starch biosynthesis, the expression of genes known to be important for tuber starch biosynthesis was investigated in leaves. Starch biosynthetic genes had a strong diurnal rhythm of expression. Interestingly, genes involved in the import of glucose 6-phosphate and ATP into the plastid are co-regulated with ADP-glucose pyrophosphorylase, suggesting that both genes are also involved in transitory starch biosynthesis in leaves (Figure 3A). To rule out that the observed expression of GPT and NTT in leaf extracts was due to contaminating epidermis rather than mesophyll cells, cell-specific RNA analysis was performed. To this end epidermis stripes were harvested and expression of GPT and NTT were probed by quantitative real-time PCR (qRT-PCR). As shown in figure 4, GPT and NTT expression in epidermis cells was about ten fold lower compared to whole leaf extracts. This supports the hypothesis that GPT and NTT are indeed required for photosynthetic starch biosynthesis in leaves. However, other cell types such as companion and bundle sheath cells could still contribute to the observed expression pattern. It can also not be excluded that the transporters are exporting substrates from the plastid.

Most starch synthases and branching enzymes had a similar pattern to the above mentioned genes in figure 3A with the exception of GBSS which was highest expressed two hours into the light period (Figure 3B). Smith *et al. *[28] argue and provide evidence that since the enzyme is present within the granule, the protein is degraded together with starch at night and must very quickly be re-synthesised in the morning. Two genes involved in the light reaction of photosynthesis, plastocyanin and chlorophyll a/b binding protein, were already up-regulated at the first time point which was taken moments after the lights came on and were highest expressed two hours into the light. Ribulose 1,5 bisphosphate carboxylase oxygenase (Rubisco) had an expression pattern similar to that of starch biosynthetic genes (Figure 3C). The sucrose cleavage enzymes cell wall-bound invertase and sucrose synthase had very different expression patterns with Susy 4 being much stronger regulated. Susy 4 increased during the light and went down in the dark. Hexokinase 1 was not diurnally regulated, whilst hexokinase 2 and fructokinase had a similar pattern of increasing early in the morning and declining at the end of the light period (Figure 3D).

**Figure 5:**
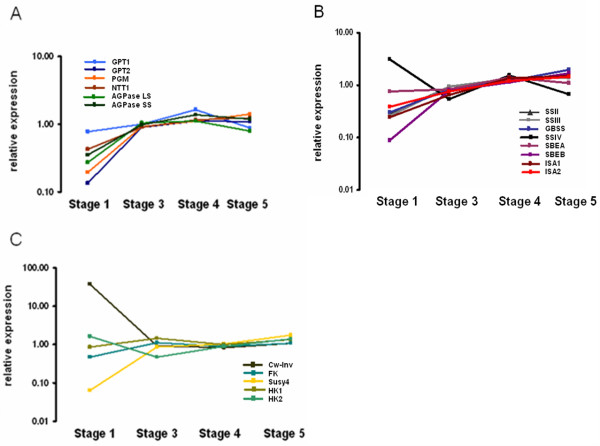
**Expression of genes known to be involved in starch biosynthesis during tuber induction**. A) Import of glucose 6-phosphate and ATP into the plastid and the conversion thereof to ADP-Glucose. GPT1 (light blue), GPT2 (dark blue), PGM (orange), NTT1 (brown), AGPase LS (light green), AGPase SS (dark green). B) Starch synthases and branching enzymes. SSII (dark grey), SSIII (light grey), GBSS (blue), SSIV (black), SBEA (light purple), SBEB (dark purple), ISA1 (dark red) and ISA2 (pink). C) Sucrose cleavage and phosphorylation. Cw-Inv (Black), FK (blue), Susy (gold), HK1 (Dark yellow) and HK2 (green). Values are the mean of two replicates.

For analysis of gene expression during tuber induction, tuber developmental stages were designated according to Kloosterman *et al. *[26] and the expression of genes were studied by microarray analysis. Stage 1, representing an unswollen stolon, showed very low expression for all starch biosynthetic genes except starch synthase IV which was highest expressed at stage 1, fitting with its proposed role in starch granule initiation [22,23]. From stages 3-5 there was an increase in the expression of all starch biosynthetic genes with the exception of starch synthase IV (Figure 5A and 5B). As evidence that stage 1 was before the onset of tuberisation, cell wall-bound invertase expression was very high and sucrose synthase 4, a tuber expressed isoform, low. From stages 3-5 the expression cell wall-bound invertase went down and that of Susy 4 increased (Figure 5C). The expression patterns compared well to earlier experiments [29]

**Figure 6:**
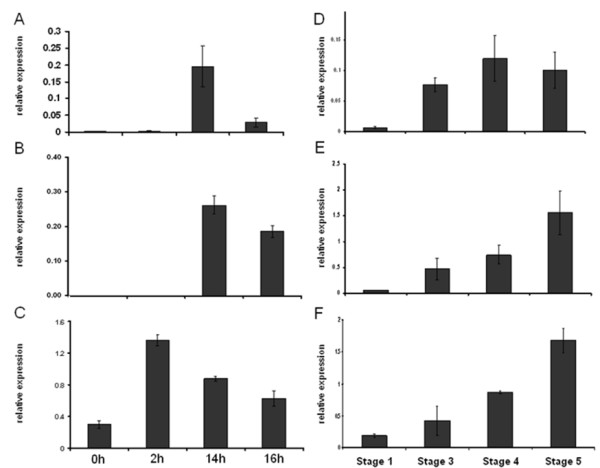
**Quantitative real time PCR confirmation of microarray results**. A-C) Relative expression of GPT2, Susy4 and GBSS diurnally in leaves. D-F) Relative expression of GPT2, Susy4 and GBSS in tubers. Error bars represent standard deviation (n = 3).

qRT-PCR is a well accepted method for verifying microarray data and was used to validate the expression patterns of a selected number of genes in independent samples. The expression patterns and levels of Susy 4, GPT and GBSS compared well to the microarray data in both leaves and tubers and confirmed the accuracy of the microarray results (Figure 6).

**Figure 7:**
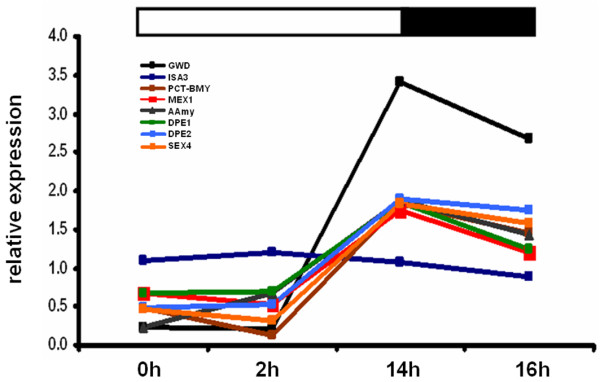
**Diurnal expression of genes known to be involved in starch degradation**. GWD (black), DPE1 (green), DPE2 (light blue), MEX1 (red), Alpha amylase (grey), Isoamylase 3 (dark blue), PCT-BMY (brown) and SEX4 (orange). Values are the mean of two replicates.

In contrast to starch biosynthesis, the pathway of starch degradation in potato tubers is not well understood. Currently it is believed that the major pathway of starch degradation in Arabidopsis and other organisms proceeds via ί-amylase [32]. Starch must first be phosphorylated by glucan-water dikinase (GWD), and to a lesser extent phospho-glucan water dikinase [33,34]. It is then either directly degraded to maltose by ί-amylase or first to linear glucans by Isoamylase 3. Before complete hydrolysis of phosphorylated glucans can occur, phosphate groups must be removed by specific a phosphatase, known as SEX4 phosphoglucan phosphatase [35]. The linear glucans are then hydrolysed to maltose by ί-amylase. Maltose is subsequently exported to the cytosol via the maltose transporter (MEX1) [36] and it is believed that the disproportioning enzyme 2 (DPE2) plays an important role in its further degradation [37]. Maltose can also be degraded by disproportioning enzyme 1 (DPE1) inside the plastid. For potato evidence has been provided that DPE2 might be present inside the plastid [38], which questions the role of MEX1. If the expression data is compared to that of Arabidopsis, indications are there that a similar pathway for starch degradation probably occurs in potato leaves, except that isoamylase 3 was not differentially expressed. The other genes mentioned were all differentially co-expressed in leaves, and expression patterns followed that of starch accumulation in the leaf (Figure 7). The entire microarray dataset for both the diurnal leaf experiment and the tuber induction experiment have been deposited on ArrayExpress (accession numbers E-MEXP-2481 Ferreira et al. Potato diurnal leaf time-course and E-MEXP-2482 Ferreira et al. Potato tuber induction). POCI sequence and annotation data are available through the POCI online tool http://pgrc.ipk-gatersleben.de/poci.

### Diurnal oscillation of GBSS in potato tubers can be linked to differences in sucrose supply

It is known that starch biosynthetic genes are diurnally regulated by several factors, with sucrose and the circadian clock seemingly being the most important. Bläsing *et al*. [39] showed that between 30-50% of genes in Arabidopsis rosettes show diurnal changes in their transcripts and that this was especially true for genes involved in redox regulation, nutrient acquisition and assimilation, and starch and sucrose metabolism. Comparative analysis of nutrient feeding and diurnal transcription profiles indicate that sugars make a major contribution to diurnal regulation. Furthermore, Osuna *et al. *[40] analysed gene expression in carbon-deprived Arabidopsis seedlings after the addition of sucrose. Genes involved in central carbon metabolism, and more specifically starch biosynthesis, showed a response to sucrose and this did lead to an increase in starch content. A second major regulator of diurnal gene expression seems to be the circadian clock. Bläsing *et al*. [39] identified a subset of 373 genes known to be circadian regulated [41]. The gene set was used in a principle component analysis which showed that sucrose and the circadian clock are the predominant factors in regulating diurnal gene expression and that light, nitrogen and water deficiency makes a smaller contribution. Starch degradation related genes also have a strong diurnal rhythm in Arabidopsis which was maintained under continuous light, but not continuous darkness [42].

**Figure 8:**
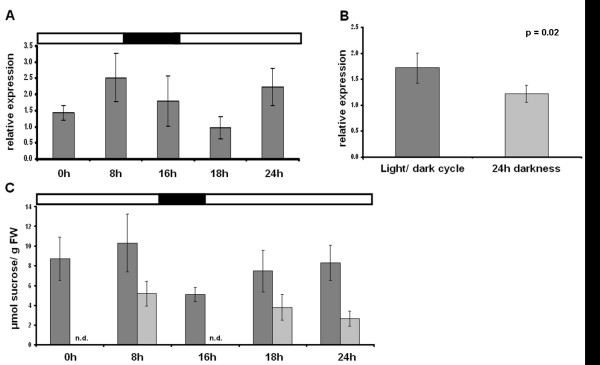
**GBSS relative expression and stolon sucrose content at different time-points of the day**. A) Diurnal expression of GBSS in tubers. B) GBSS expression at the 24 hour time-point from plant grown in light/dark cycle and from plants kept in twenty four hours of darkness. C) Stolon sucrose content at different time-points of the day (dark grey bars) and at the same time-points from plant kept in darkness from 0 hours onward (dark grey bars). Two values were not determined (n.d.). Error bars represent standard deviation (n = 3-7).

Since GBSS is one of the strongest diurnally regulated genes involved in starch biosynthesis [28], it was investigated whether this gene is also diurnally regulated in tubers. GBSS expression was measured at different times of the day and under different light regimes. Plants were grown in normal long day conditions and samples were taken from stolon-ends and tubers at different time-points of the diurnal cycle. GBSS expression in tubers oscillated during the day and was highest at the end of the light period and lowest 2 hours into the next light period (Figure 8A). Expression was also significantly lower in tubers from plants kept in twenty four hours of darkness compared to tubers from plants kept in a normal light/dark cycle (Figure 8B).

As mentioned, sucrose and the circadian clock seem to be important regulators of diurnal gene expression and this is especially true for GBSS. Tenioro *et al*. [43] showed that GBSS is strongly regulated by the circadian clock and that expression is markedly lower in mutants lacking clock genes LHY and CCA-OX respectively. In a detailed study done on GBSS in snapdragon (*Antirrhinum majus*) it was shown that GBSS is diurnally regulated in leaves even under continuous light and it was concluded that the regulation is due to the circadian clock. This was not the case in snapdragon roots though, where expression was the same in the middle of the day and in the middle of the night [44]. Also in rice leaves GBSS continues its diurnal cycling under continuous light suggesting circadian regulation, but expression can be induced by nitrogen starvation or sucrose feeding and repressed by darkness, indicating the importance of sucrose in its regulation [45]. Moreover, sucrose floating experiments with potato leaves show that GBSS expression can be induced by sucrose [46].

To investigate whether oscillation of GBSS might be due to changes in sucrose supply from the source, sucrose content of stolon-ends attached to a tuber was measured. Phloem sucrose content is very high compared to those tissues surrounding it [47] and phloem signifies a large proportion of the total stolon tissue [48]. This makes it possible to determine sucrose import to the tuber by measuring sucrose content of the stolon-end [3]. Sucrose content differed significantly during the day and was highest at the end of the light and lowest at the end of the dark period. When plants were kept in constant darkness, sucrose content of stolon-ends declined linearly over time indicating that changes in sucrose supply from the leaves contributes to the oscillating expression of GBSS in tubers (Figure 8C).

Although GBSS expression in tubers can be linked to diurnal changes in sucrose supply from the source, caution should be exercised in the interpretation of the significance of this in terms of enzyme activity. As mentioned earlier, the level of GBSS protein does change substantially during the day in Arabidopsis leaves and the reason for this is probably the location of GBSS within the granule [28]. This is also true for algae *Chlamydomonas reinhardtii *suspension cultures where GBSS expression and enzyme activity correlates with starch levels. The authors state that the correlation is probably due to the fact that the analysis was conducted in suspension cultures, where new cells are produced constantly leading to continuous production of new GBSS protein [49]. This however is not necessarily true for GBSS in other tissues or for other enzymes of starch biosynthesis and several studies have shown that diurnal changes in expression do not lead to changes in protein levels [9,42]. However, it still remains an interesting finding that GBSS expression in tubers follows a diurnal rhythm which declines when sucrose supply from the leaves are reduced.

### Starch biosynthetic gene expression is influenced by tuber growth velocity

**Figure 9:**
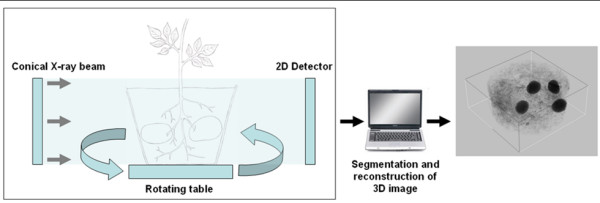
**Schematic scheme illustrating how tuber volume is measured using X-ray CT**. Potato plants are scanned with X-ray in a chamber containing an X-ray beam and a two dimensional detector. After projecting X-ray images of the potato plant on the detector at different angles, the projections are reconstructed *in silico *to create a three dimensional image. From this image the tuber volume can be calculated.

**Figure 10:**
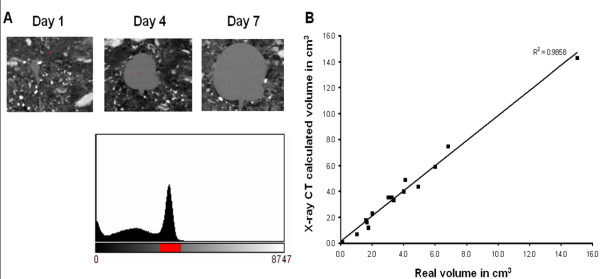
**Potato tuber segmentation and volume calculation**. A) Two dimensional X-ray images illustrating that potato tubers can be distinguished from the surrounding soil. The histogram shows that segmentation is possible. The red bar indicates the grey level threshold selected. B) Linear regression of X-ray calculated and real volume measurements confirming the accuracy of X-ray CT calculated volumes.

As mentioned earlier, tuber initiation and growth rates are not synchronised. Because analysis of tubers growing at different velocities would provide important information on factors determining growth, it was thought necessary to develop a method to determine growth velocity before harvesting the tubers. For this, X-ray CT was used to determine tuber volume over a time course and then calculate the growth velocity. An overview of the method for calculating tuber volume using X-ray CT is given in figure 9. The accuracy of the growth velocity determination depends largely on the accuracy of volume calculation and it was crucial to establish whether tuber volume could be accurately calculated. Figure 10A illustrate that potato tubers were clearly distinguishable from the surrounding soil, and a histogram demonstrated that segmentation of tuber material was possible. The software used for segmentation also allowed for manual correction of possible mistakes made during the automatic segmentation process. Tuber growth velocities were calculated for seventeen tubers. To validate the method, calculated tuber volumes were compared to measured tuber volumes after harvest. X-ray CT calculated volumes showed a correlation coefficient with experimental volume measurements of 0.986 showing that X-ray CT calculated volumes were accurate (Figure 10B).

**Figure 11:**
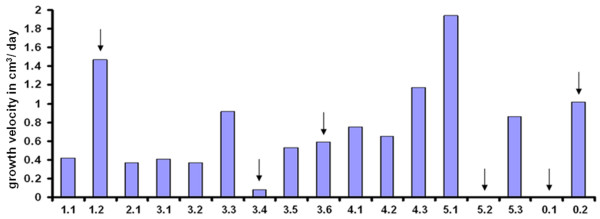
**Estimated growth velocity of tubers in cubic centimetre volume increase per day**. Tubers chosen for microarray hybridisation are marked by arrow heads

**Figure 12:**
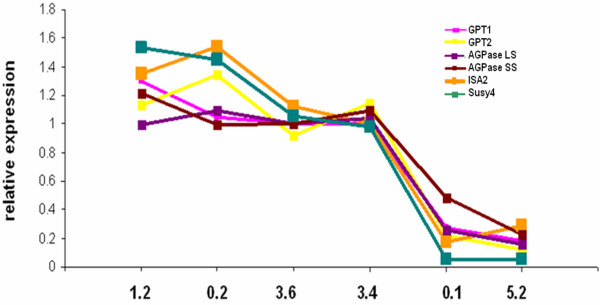
**Relative expression of starch biosynthetic genes in tubers growing at different velocities**. GPT1 (pink), GPT2 (yellow), ISA2 (orange), AGPase LS (purple), AGPase SS (brown) and Susy4 (green). Values are the mean of two replicates.

Six tubers with different growth velocities (Figure 11) were selected for microarray hybridisation and the gene expression patterns were analysed. Interestingly no linear correlation between the expression of starch biosynthetic genes and growth velocity could be observed. The expression of genes involved in starch biosynthesis is shown in figure 12. The expression of these genes, and also other genes involved in starch biosynthesis, did not show large differences in expression levels between tubers that were still growing, albeit at very different velocities. Starch biosynthetic gene expression was however much lower in tubers that have virtually stopped growing and it seems as though the relationship between growth velocity and starch biosynthetic gene expression is qualitative rather than quantitative. This was especially true for Susy, a major determinant of tuber sink strength [11].

These data indicate that tubers that look visually similar have large differences in gene expression depending on their growth stage. Moreover, transcription profiles reveal many genes that are differentially expressed between growing and non-growing tubers which provide important information towards the identification of factors determining tuber growth. The entire microarray dataset for the tuber growth velocity experiment has been deposited on ArrayExpress (accession number E-MEXP-2484 Ferreira et al. tuber growth velocity).

### Comparative analysis of transcription profiles reveals genes possibly regulating starch biosynthesis

**Figure 13:**
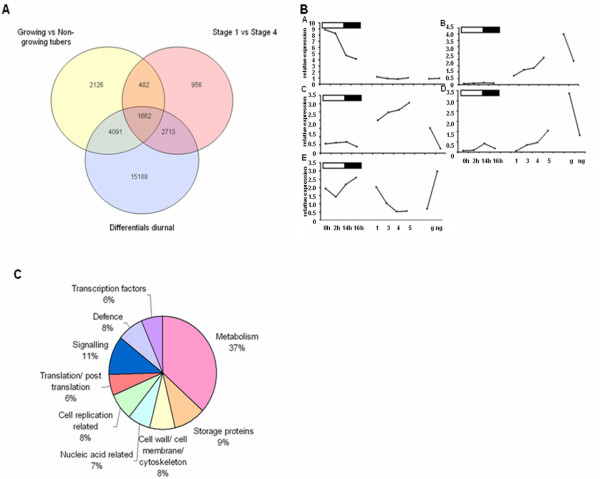
**Comparative analysis of transcription profiles**. A) Venn diagramm showing 1662 features differentially expressed under all conditions selected B) K means clustering showing 5 clusters (A-E) with three clusters having the desired pattern of expression for starch biosynthetic genes (B-D). C) Functional assignment of features present in clusters B-D).

The similarities observed in terms of starch biosynthetic gene expression between leaves and tubers suggests that the same regulators might be involved in both processes. In an attempt to identify these regulators, comparative analyses of various transcription profiles were conducted. Firstly, features differentially expressed between a stolon/swollen stolon, diurnally in leaves and between growing/non-growing tubers were selected. To further decrease the number of features, only those features differentially expressed under all conditions were selected which reduced the number to 1662 (Figure 13A). K-means clustering analysis was performed on these features and most starch biosynthetic genes were present in three clusters. These clusters also showed the expected expression patterns for starch biosynthetic genes (Figure 13B). A functional assignment on the 913 features present in these three clusters was performed which showed that 37% of the features were involved in metabolism and a further 9% storage protein related (Figure 13C). Transcription factors made up 6% of the features and these were further investigated.

**Figure 14:**
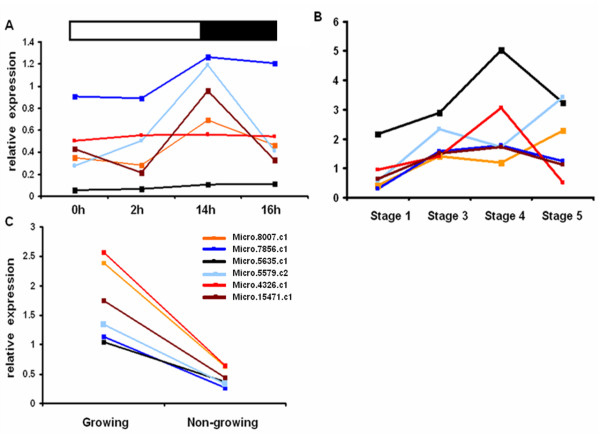
**Expression profiles of transcription factors possibly regulating starch biosynthesis**. A) Diurnally in leaves, B) during tuber induction and C) growing and non-growing tubers. Micro.8007 (AT2g40820), orange, Micro.7865.c1 (AT3G16280), blue, Micro.5635.c1 (AT4G37750), black, Micro.5579.c2 (AT4G34590), light blue, Micro.15471.c1 (AT4G00870), brown and Micro.4326.c1 (AT4G32730), red.

Arabidopsis orthologs for these transcription factors were identified and used to gather more information on their possible function. Six of these transcription factors showed increased expression in Arabidopsis suspension culture fed with sucrose [50] and most of these genes were also down-regulated in leaves when the dark period was extended [51]. Figure 14 contain the expression profiles of these transcription factors including the identifiers of their orthologs in Arabidopsis. All of these transcription factors, with the exception of AT4G00870 and AT4G32730, were also down-regulated in the 35S::amiR-white-1(MIR172a) knockout [52]. The construct targets the GUN4 allele which is important for plastid to nucleus signalling and when this is interrupted it affects plastidic expression of several nuclear encoded genes. Furthermore, the expression of GPT2, plastidial PGM, GBSS and SBEB was also significantly down in these plants. This, along with the fact that these transcription factors were co-expressed in the same clusters as starch biosynthetic genes, indicate that they might be important in regulating starch accumulation.

## Conclusion

Comparative transcriptome analysis between leaves and tubers indicate that transient and storage starch biosynthesis might not be all that different with the same isoforms being differentially expressed in both tissues. There was also a diurnal rhythm of GBSS expression in tubers which could be correlated to sucrose supply from the leaves. This provided evidence not only of the diurnal regulation of starch biosynthetic gene expression in tubers, but also showed the importance of sucrose supply in regulating gene expression in tubers.

Since tuber initiation and growth is not synchronised, it was important to determine the growth velocity of individual tubers. To this end X-ray CT was used to determine the volume of individual tubers at different time points and calculate the growth velocity. This was the first time that the growth velocities of tubers were determined in a natural environment. Tuber growth velocity could not be correlated to starch biosynthetic gene expression, although it was clear that gene expression is different between growing and non-growing tubers. The relationship between gene expression and growth velocity seems to be qualitative rather than quantitative and the data provides important information towards the identification of factors determining potato tuber growth.

Comparative analysis made it possible to select for genes differentially regulated under various conditions. Since the microarray experiments performed were set up to comprise conditions of active starch biosynthesis, it was believed that comparative analysis of transcription profiles would select for genes involved in this process. Cluster analysis of differentially expressed genes revealed clusters containing genes known to be involved in starch biosynthesis, and further analysis revealed transcription factors which could be used to influence starch biosynthesis. Closer analysis reveal that orthologs of these transcription factors in Arabidopsis are positively regulated by sucrose indicating that they could be interesting targets for influencing starch biosynthesis.

## Methods

### Plants and growth conditions

*Solanum tuberosum (cv Solara) *were propagated in tissue culture on MS medium [53] containing 2% sucrose. To obtain tubers, plants were transferred to soil and cultivated until harvest in individual pots in the greenhouse or growth chambers. For transcriptional analysis of potato leaves over a diurnal period, plants were grown in a growth chamber under a 14 hour light and 10 hour dark cycle. Plants for the tuber induction and X-ray CT studies were grown in the greenhouse under normal long day conditions. Plants used for analysing for the diurnal rhythm in tuber gene expression were grown in growth chambers under normal long day conditions.

### RNA isolation, cRNA synthesis and Cy3-labeling

Isolation of total RNA was performed as described previously [54].

### POCI array and database

The construction of the POCI array and database has been described in detail [29].

### Sample preparation and microarray hybridization

For each hybridisation at least two biological replicates were included. For diurnal leaf time-points five different leaves from five plants were sampled for each biological replicate. For tuber induction samples stolon or tuber material from 40 plants were pooled into two groups according to the developmental stages. For the hybridization comparing fast and slow growing tubers, two independent samples were taken from each tuber and treated as biological replicates. RNA purity was measured by the ND-1000 Spectrophotometer (NanoDrop Technologies). To check for RNA degradation two μg of total RNA were separated on 1.5% formaldehyde containing agarose gel. Total RNA was purified using RNeasy Mini Spin Columns (QIAGEN, Valencia, CA). Afterwards RNA quality and quantity was tested using the Agilent 2100 BioAnalyzer (vB.02.03 BSI307) as recommended by manufacturer's protocol (Agilent RNA 6000 Nano Assay Protocol2). Synthesis of cDNA and cRNA was performed as described in the one-color microarray-based gene expression analysis protocol provided by Agilent including the one-color RNA spike-in kit (v5.0.1, 2006; Agilent Technologies, Santa Clara). After fragmentation Cy3-labelled samples were loaded on the array and hybridised over night (17h/65°C). Slides were washed as recommended in the manufacturer's protocol and scanned on the Agilent Microarray Scanner with extended dynamic range (XDR) at high resolution (5 μm). Data sets were extracted by using the feature extraction software (v9.5.3.1/Agilent Technologies) using a standard protocol.

### Array data analysis

Data were imported into GeneSpring GX 7.3.1 (Silicon Genetics, Palo Alto, CA, USA) and additionally stored on a local server. A three step normalization was applied: (1) values less than five were set to five, (2) per chip normalization to 50^th ^percentile and (3) subsequently the signal for each feature was normalised to the median of its value across the entire dataset. For comparative analysis of various transcription profiles, a volcano plot was applied to select for features more than two-fold differentially expressed between two conditions including the Benjamini-Hochberg multiple test correction for the four replicate experiment comparing slow and fast growing tubers. No multiple test correction was employed for the experiments comparing stolon and swollen stolon, and the diurnal leaf experiment, where only two replicates per data-point were used. Features commonly differentially expressed in all experiments were identified using a Venn diagram. K-means clustering was performed using Pearson correlation to split the selected features into five clusters from where features were chosen to conduct a functional assignment.

### Starch and sucrose measurements

Sucrose and starch were measured according to a modified method of Müller-Röber *et al *[17]. To determine sucrose supply rate to a tuber, sucrose content of stolon-ends was determined. A stolon-end is defined as the 15 millimetres of a stolon directly above the connection to the tuber.

### qPCR analysis

For all qPCR analysis at least three biological repeats were used unless stated otherwise. Expression levels of genes were determined by real-time quantitative RT-PCR and the corresponding primers for the amplification of targets between 75 and 150 bp were designed using Primer3plus software [55]. Total RNA (five μg) from each of the developmental time points was treated with DNaseI (Fermentas GmbH) before undergoing reverse transcription using oligo d(T) primers and RevertAid™ H minus first strand cDNA synthesis kit (Fermentas GmbH) to generate a first strand cDNA template. Potato ubiquitin primers were used as a control as described previously [26]. One μl of 1:10 diluted cDNA for each time point were amplified with gene-specific primers in three technical replicates on a Mx3000P Q-PCR system (Stratagene) in combination with the Brilliant II SYBR Green Q-PCR Master Mix Kit (Stratagene). The thermal profile was as follows: 1 cycle 10 min at 95°C for DNA polymerase activation followed by 35 cycles of 10 s at 95°C, 15 s 60°C and 20 s 72°C. The primer sequences were as follows: *ubi3 *(L22576) forward primer, 5'-TTCCGACACCAT CGACAATGT-3'; reverse primer, 5'-CGACCATCCTCAAGCTGCTT-3'. For GBSS the primer sequence was based on POCI feature micro.920.c2. The forward primer was designated GBSS_920.c2 F (5' - CAGACTTGAGGAGCAGAAAGG - 3') and the reverse primer GBSS_920.c2 R (5' - GTGAGCCAAAGGGACATTGA - 3'). For GPT the primer sequence was based on POCI feature micro.1076.c1. The forward primer was designated GPT_1076.c1 F (5' -CCTTGTTTCCTGTTGCTGTG- 3') and the reverse primer GBSS_1076.c1 R (5' -AAAGCAGGCTCTCCACTCTT- 3'). For Susy the primer sequence was based on POCI feature micro.196.c8. The forward primer was designated Susy_196.c8 F (5' -CTGCTGTTTATGGGTTCTGG- 3') and the reverse primer Susy_196.c1 R (5' -GGCACACCTTCATTCACTCA- 3'). For NTT the primer sequence was based on POCI feature micro.1831.c2. The forward primer was designated NTT_1831.c2 F (5' -GAGCAGCAGCCAAGATAACAC- 3') and the reverse primer GBSS_1831.c2 R (5' -GTTCTGCATTGCACCCACA- 3'). Relative gene expression was calculated using the Pfaffl method [56].

### Description of X-ray CT

With X-ray CT the 3D volume information of objects can be reconstructed using X-ray projections of the object from different aspects. The geometry used for the investigation was the axial 3D-CT, where a conical X-ray beam projects the object onto a flat 2D image detector. Using axial 3D-CT, projections of the object are taken under different viewing angles, rotating the object perpendicular to the central X-ray beam. The reconstructed volume data set consists of volumetric elements, called voxels, containing grey levels which represent information about the X-ray attenuation characteristics depending on the mass attenuation coefficient and the density distribution of the material [57,58]. The mass attenuation coefficient itself is dependent on the applied X-ray spectrum and the effective atomic number of the X-rayed material. The calculation of tuber volumes of potted potato plants embedded in soil requires the segmentation of tubers from other materials in the X-ray CT volume data. Therefore a careful selection of exposure conditions is necessary to achieve sufficient data quality. This comprises X-ray parameters and filters as well as the condition of the soil. The parameters were defined as such: The X-ray source was FXE-225.45, accelerating voltage 200 kV, total emission 200 μA, the filter 1 mm Cu, detector Perkin Elmer, 1024 × 1024 pixel 200 μm, scan period 50 minutes and a resolution of 141 μm. After segmentation voxel elements, which have a known volume and specific grey level for tubers, were used to determine tuber volume by calculating how many voxels are present in a reconstructed tuber image. The lower and upper threshold grey values were set at 2938 and 3963 after which mistakes were corrected for manually. Tuber volume calculations were performed using Image J software http://rsbweb.nih.gov/ij.

## Authors' contributions

SJF did most of the experimental work and also drafted the manuscript. MS helped with the tuber volume calculations, microarray data analysis and functional assignment of features. SS helped with microarray hybridisations and data analysis. PK supervised and RG initiated the X-ray CT work. US provided the project funding, conceived and led the study and significantly contributed to drafting the manuscript. All authors read and approved the final manuscript.

## References

[B1] Kruger NJ Dennis DT, Turpin DH, Lefebvre DD, Layzell DB Carbohydrate synthesis and degradation Plant Metabolism 1997 Harlow: Longman 83 104

[B2] Farrar JF Pollock CJ, Farrar JF, Gordon AJ The whole plant: Carbon partitioning during development Carbon Partition within and Between Organisms 1992 Oxford: Bios Scientific publishers 163 179

[B3] Sweetlove LJ Kossmann J Riesmeier JW Trethewey RN Hill SA The control of source to sink carbon flux during tuber development in potato Plant J 1998 15 697 706 10.1046/j.1365-313x.1998.00247.x 29368807

[B4] Zrenner R Krause KP Apel P Sonnewald U Reduction of the cytosolic fructose-1,6-bisphosphatase in transgenic potato plants limits photosynthetic sucrose biosynthesis with no impact on plant growth and tuber yield Plant J 1996 9 671 681 10.1046/j.1365-313X.1996.9050671.x 8653116

[B5] Riesmeier JW Willmitzer L Frommer WB Evidence for an essential role of the sucrose transporter in phloem loading and assimilate partitioning EMBO J 1994 13 1 7 8306952 10.1002/j.1460-2075.1994.tb06229.x PMC394773

[B6] Kühn C Quick WP Schulz A Riesmeier JW Sonnewald U Frommer WB Companion cell-specific inhibition of the potato sucrose transporter SUT1 Plant Cell Enviro 1996 19 1115 1123 10.1111/j.1365-3040.1996.tb00426.x

[B7] Leggewie G Kolbe A Lemoine R Roessner U Lytovchenko A Zuther E Kehr J Frommer WB Riesmeier JW Willmitzer L Fernie AR Overexpression of the sucrose transporter SoSUT1 in potato results in alterations in leaf carbon partitioning and in tuber metabolism but has little impact on tuber morphology Planta 2003 217 158 167 12721860 10.1007/s00425-003-0975-x

[B8] Sweetlove LJ Hill SA Source metabolism dominates the control of source to sink carbon flux in tuberizing potato plants throughout the diurnal cycle and under a range of environmental conditions Plant Cell Environ 2000 23 523 529 10.1046/j.1365-3040.2000.00567.x

[B9] Geigenberger P Stitt M Diurnal changes in sucrose, nucleotides, starch synthesis and AGPS transcript in growing potato tubers that are suppressed by decreased expression of sucrose phosphate synthase Plant J 2000 23 795 806 10.1046/j.1365-313x.2000.00848.x 10998190

[B10] Viola R Roberts AG Haupt S Gazzani S Hancock RD Marmiroli N Machray GC Oparka KJ Tuberization in potato involves a switch from apoplastic to symplastic phloem unloading Plant Cell 2001 13 385 1398 10.1105/tpc.13.2.385 11226192 PMC102249

[B11] Zrenner R Salanoubat M Willmitzer L Sonnewald U Evidence of the crucial role of sucrose synthase for sink strength using transgenic potato plants (*Solanum tuberosum L.*) Plant J 1995 7 97 107 10.1046/j.1365-313X.1995.07010097.x 7894514

[B12] Fettke J Albrecht T Hejazi M Mahlow S Nakamura Y Steup M Glucose 1-phosphate is efficiently taken up by potato (*Solanum tuberosum*) tuber parenchyma cells and converted to reserve starch granules New Phytol 2009 185 3 663 675 10.1111/j.1469-8137.2009.03126.x 20028468

[B13] Kammerer B Fischer K Hilpert B Schubert S Gutensohn M Weber A Flügge UI Molecular Characterization of a Carbon Transporter in Plastids from Heterotrophic Tissues: The Glucose 6-Phosphate/Phosphate Antiporter Plant Cell 1998 10 105 118 10.1105/tpc.10.1.105 9477574 PMC143937

[B14] Tjaden J Möhlmann T Kampfenkel K Henrichs G Neuhaus HE Altered plastidic ATP/ADP-transporter acitivity influences potato (*Solanum tuberosum L.*) tuber morphology, yield and composition of tuber starch Plant J 1998 16 531 540 10.1046/j.1365-313x.1998.00317.x

[B15] Zhang L Häusler RE Greiten C Hajirezaei MR Haferkamp I Neuhaus HE Flügge UI Ludewig F Overriding the co-limiting import of carbon and energy into tuber amyloplasts increases the starch content and yield of transgenic potato plants Plant Biotechnol J 2008 6 453 464 10.1111/j.1467-7652.2008.00332.x 18363632

[B16] Tauberger E Fernie AR Emmermann M Renz A Kossmann J Willmitzer L Trethewey RN Antisense inhibition of plastidial phosphoglucomutase provides compelling evidence that potato tuber amyloplasts import carbon from the cytosol in the form of glucose-6-phosphate Plant J 2000 23 43 53 10.1046/j.1365-313x.2000.00783.x 10929100

[B17] Müller-Röber B Sonnewald U Willmitzer L Inhibition of the ADP-glucose pyrophosphorylase in transgenic potatoes leads to sugar-storing tubers and influences tuber formation and expression of tuber storage protein genes EMBO J 1992 11 1229 1238 1373373 10.1002/j.1460-2075.1992.tb05167.x PMC556571

[B18] Stark DM Timmerman KP Barry GF Preiss J Kishore GM Regulation of the Amount of Starch in Plant Tissues by ADP Glucose Pyrophosphorylase Science 1992 258 287 292 10.1126/science.258.5080.287 17835129

[B19] Sweetlove LJ Burrell MM ap Rees T Starch metabolism in tubers of transgenic potato (*Solanum tuberosum*) with increased ADP glucose pyrophosphorylase Biochem J 1996 320 493 498 8973558 10.1042/bj3200493 PMC1217957

[B20] Lloyd JR Landschütze V Kossmann J Simultaneous antisense inhibition of two starch-synthase isoforms in potato tubers leads to accumulation of grossly modified amylopectin Biochem J 1999 338 515 521 10.1042/0264-6021:3380515 10024530 PMC1220080

[B21] Hovenkamp-Hermelink JHM Jacobsen E Ponstein AS Visser RGF Vos-Scheperkeuter GH Bijmolt EW De Vries JN Witholt B Feenstra WJ Isolation of an amylose-free starch mutant of the potato (*Solanum tuberosum *L.) Theor Appl Genet 1987 75 217 221 10.1007/BF00249167

[B22] Roldán I Wattebled F Mercedes Lucas M Delvallé D Planchot V Jiménez S Pérez R Ball S D'Hulst C Mérida A The phenotype of soluble starch synthase IV defective mutants of Arabidopsis thaliana suggests a novel function of elongation enzymes in the control of starch granule formation Plant J 2007 49 492 504 10.1111/j.1365-313X.2006.02968.x 17217470

[B23] Szydlowski N Ragel P Raynaud S Lucas MM Roldán I Montero M Muñoz FJ Ovecka M Bahaji A Planchot V Pozueta-Romero J D'Hulst C Mérida A Starch granule initiation in Arabidopsis requires the presence of either class IV or class III starch synthases Plant J 2009 21 2443 2457 10.1105/tpc.109.066522 PMC2751949 19666739

[B24] Kossmann J Visser RG Müller-Röber B Willmitzer L Sonnewald U Cloning and expression analysis of a potato cDNA that encodes branching enzyme: evidence for co-expression of starch biosynthetic genes Mol Gen Genet 1991 230 39 44 10.1007/BF00290648 1745241

[B25] Schwall GP Safford R Westcott RJ Jeffcoat R Tayal A Shi YC Gidley MJ Jobling SA Production of very-high-amylose potato starch by inhibition of SBE A and B Nat Biotechnol 2000 18 551 554 10.1038/75427 10802625

[B26] Kloosterman BA Vorst OFJ Hall RD Visser RGF Bachem CWB Tuber on a chip: differential gene expression during potato tuber development Plant Biotechnol J 2005 505 519 10.1111/j.1467-7652.2005.00141.x 17173637

[B27] Han L Dutilleul P Prasher SO Beaulieu C Smith Assessment of common scab-inducing pathogen effects on potato underground organs via computed tomography scanning Phytopathology 2008 98 1118 1125 10.1094/PHYTO-98-10-1118 18943458

[B28] Smith SM Fulton DC Chia T Thorneycroft D Chapple A Dunstan H Hylton C Zeeman SC Smith AM Diurnal changes in the transcriptome encoding enzymes of starch metabolism provide evidence for both transcriptional and posttranscriptional regulation of starch metabolism in Arabidopsis leaves Plant Physiol 2004 136 2687 2699 10.1104/pp.104.044347 15347792 PMC523333

[B29] Kloosterman B De Koeyer D Griffiths R Flinn B Steuernagel B Scholz U Sonnewald S Sonnewald U Bryan GJ Prat S Bánfalvi Z Hammond JP Geigenberger P Nielsen KL Visser RG Bachem CW Genes driving potato tuber initiation and growth: identification based on transcriptional changes using the POCI array Funct Integr Genomics 2008 4 329 340 10.1007/s10142-008-0083-x 18504629

[B30] Arce AL Cabello JV Chan RL Patents on plant transcription factors Recent Pat Biotechnol 2008 2 209 217 10.2174/187220808786241024 19075869

[B31] Butelli E Titta L Giorgio M Mock HP Matros A Peterek S Schijlen EG Hall RD Bovy AG Luo J Martin C Enrichment of tomato fruit with health-promoting anthocyanins by expression of select transcription factors Nat Biotechnol 2008 26 1301 1318 10.1038/nbt.1506 18953354

[B32] Scheidig A Fröhlich A Schulze S Lloyd JR Kossmann J Downregulation of a chloroplast-targeted beta-amylase leads to a starch-excess phenotype in leaves Plant J 2002 30 581 91 10.1046/j.1365-313X.2002.01317.x 12047632

[B33] Ritte G Lloyd JR Eckermann N Rottmann A Kossmann J Steup M The starch-related R1 protein is an alpha -glucan, water dikinase PNAS 2002 10 7166 7171 10.1073/pnas.062053099 PMC124546 12011472

[B34] Baunsgaard L Lütken H Mikkelsen R Glaring MA Pham TT Blennow A A novel isoform of glucan, water dikinase phosphorylates pre-phosphorylated alpha-glucans and is involved in starch degradation in Arabidopsis Plant J 2005 9 595 605 10.1111/j.1365-313X.2004.02322.x 15686522

[B35] Kötting O Santelia D Edner C Eicke S Marthaler T Gentry MS Comparot-Moss S Chen J Smith AM Steup M Ritte G Zeeman SC STARCH-EXCESS4 is a laforin-like Phosphoglucan phosphatase required for starch degradation in *Arabidopsis thaliana* Plant Cell 2009 21 334 346 10.1105/tpc.108.064360 19141707 PMC2648081

[B36] Niittylä T Messerli G Trevisan M Chen J Smith AM Zeeman SC A previously unknown maltose transporter essential for starch degradation in leaves Science 2004 303 87 89 10.1126/science.1091811 14704427

[B37] Chia T Thorneycroft D Chapple A Messerli G Chen J Zeeman SC Smith SM Smith AM A cytosolic glucosyltransferase is required for conversion of starch to sucrose in Arabidopsis leaves at night Plant J 2004 37 853 863 10.1111/j.1365-313X.2003.02012.x 14996213

[B38] Lloyd JR Blennow A Burhenne K Kossmann J Repression of a novel isoform of disproportionating enzyme (stDPE2) in potato leads to inhibition of starch degradation in leaves but not tubers stored at low temperature Plant Physiol 2004 134 1347 1354 10.1104/pp.103.038026 15034166 PMC419812

[B39] Bläsing OE Gibon Y Günther M Höhne M Morcuende R Osuna D Thimm O Usadel B Scheible WR Stitt M Sugars and circadian regulation make major contributions to the global regulation of diurnal gene expression in Arabidopsis Plant Cell 2005 17 3257 3281 10.1105/tpc.105.035261 16299223 PMC1315368

[B40] Osuna D Usadel B Morcuende R Gibon Y Bläsing OE Höhne M Günter M Kamlage B Trethewey R Scheible WR Stitt M Temporal responses of transcripts, enzyme activities and metabolites after adding sucrose to carbon-deprived Arabidopsis seedlings Plant J 2007 49 463 491 10.1111/j.1365-313X.2006.02979.x 17217462

[B41] Harmer SL Hogenesch JB Straume M Chang HS Han B Zhu T Wang X Kreps JA Kay SA Orchestrated transcription of key pathways in Arabidopsis by the circadian clock Science 2000 290 2110 2113 10.1126/science.290.5499.2110 11118138

[B42] Lu Y Gehan JP Sharkey TD Daylength and circadian effects on starch degradation and maltose metabolism Plant Physiol 2005 138 2280 2291 10.1104/pp.105.061903 16055686 PMC1183414

[B43] Tenorio G Orea A Romero JM Mérida A Oscillation of mRNA level and activity of granule-bound starch synthase I in Arabidopsis leaves during the day/night cycle Plant Mol Biol 2003 51 949 958 10.1023/A:1023053420632 12777053

[B44] Merida A Rodrıguez-Galan JM Vincent C Romero JM Expression of the Granule-Bound Starch Synthase I (Waxy) Gene from Snapdragon Is Developmentally and Circadian Clock Regulated Plant Physiol 1999 120 401 409 10.1104/pp.120.2.401 10364391 PMC59278

[B45] Dian W Jiang H Chen Q Liu F Wu P Cloning and characterization of the granule-bound starch synthase II gene in rice: gene expression is regulated by the nitrogen level, sugar and circadian rhythm Planta 2003 218 261 268 10.1007/s00425-003-1101-9 12955512

[B46] Visser RG Stolte A Jacobsen E Expression of a chimaeric granule-bound starch synthase-GUS gene in transgenic potato plants Plant Mol Biol 1991 17 691 699 10.1007/BF00037054 1912493

[B47] Lohaus G Winter H Riens B Heldt HW Further studies of the phloem loading process in leaves of barley and spinach - the comparison of the metabolite concentration in the apoplastic compartment with those in the cytosolic compartment and in the sieve tubes Bot Acta 1995 108; 270 275

[B48] Engels CH Marschner H Allocation of photosynthate to individual tubers of *Solanum tuberosum *L. II. **Relationship between growth rate, carbohydrate concentration and **^**14**^**C-partitioning within tubers** J Exp Bot 1986 37 1804 1812 10.1093/jxb/37.12.1804

[B49] Ral JP Colleoni C Wattebled F Dauvillée D Nempont C Deschamps P Li Z Morell MK Chibbar R Purton S d'Hulst C Ball SG Circadian clock regulation of starch metabolism establishes GBSSI as a major contributor to amylopectin synthesis in *Chlamydomonas reinhardtii* Plant Physiol 2006 142 305 317 10.1104/pp.106.081885 16844835 PMC1557617

[B50] Rogers LA Dubos C Cullis IF Surman C Poole M Willment J Mansfield SD Campbell MM Light, the circadian clock, and sugar perception in the control of lignin biosynthesis J Exp Bot 2005 56 1651 1663 10.1093/jxb/eri162 15878986

[B51] Usadel B Bläsing OE Gibon Y Retzlaff K Höhne M Günther M Stitt M Expression data of Arabidopsis thaliana rosettes in an extended night http://mapman.mpimp-golm.mpg.de/supplement/xn/

[B52] Schwab R Ossowski S Riester M Warthmann N Weigel D Highly Specific Gene Silencing by Artificial MicroRNAs in *Arabidopsis* Plant Cell 2006 18 1121 1133 10.1105/tpc.105.039834 16531494 PMC1456875

[B53] Murashige T Skoog F A revised medium for rapid growth and bioassays with tobacco tissue culture Physiol Plant 1962 15 473 497 10.1111/j.1399-3054.1962.tb08052.x

[B54] Logemann J Schell J Willmitzer L Improved method for the isolation of RNA from plant tissues Anal Biochem 1987 163 16 20 10.1016/0003-2697(87)90086-8 2441623

[B55] Untergasser A Nijveen H Rao X Bisseling T Geurts R Leunissen JA Primer3Plus, an enhanced web interface to Primer3 Nucleic Acids Res 2007 35 Web Server W71 74 10.1093/nar/gkm306 17485472 PMC1933133

[B56] Pfaffl MW A new mathematical model for relative quantification in real-time PT-PCR Nucleic Acid Res 29 2002 2007 10.1093/nar/29.9.e45 PMC55695 11328886

[B57] Feldkamp LA Davis L Kress J Practical Cone-beam Algorithm J Opt Soc Am 1984 1 612 619 10.1364/JOSAA.1.000612

[B58] Hanke R Fuchs T Uhlmann N X-ray based methods for non-destructive testing and material characterization Nucl Instr and Meth in Phys Res A 2008 591 14 18 10.1016/j.nima.2008.03.016

